# Differential spleen immune signatures and germinal center responses during acute infection with *Orientia tsutsugamushi* Karp versus Gilliam strains

**DOI:** 10.3389/fimmu.2026.1791779

**Published:** 2026-04-23

**Authors:** Casey Gonzales, Yuejin Liang, Joseph Thiriot, Hui Wang, Spencer Reiling, Dario Villacreses, Jiaren Sun, Lynn Soong

**Affiliations:** 1 Department of Pathology, University of Texas Medical Branch, Galveston, TX, United States; 2 Department of Microbiology and Immunology, University of Texas Medical Branch, Galveston, TX, United States; 3 Institute of Human Infections and Immunity, University of Texas Medical Branch, Galveston, TX, United States

**Keywords:** B cell, germinal center, *Orientia tsutsugamushi*, scrub typhus, splenic architecture

## Abstract

**Introduction:**

Scrub typhus is an emerging and neglected tropical disease caused by *Orientia tsutsugamushi* (*Ot*). Immunity in scrub typhus patients is known to be short-lived; however, its underlying mechanisms remain unclear. No reports have examined humoral immune signatures to clinically prevalent *Ot* strains.

**Methods:**

We compared two clinically relevant Ot strains, Karp and Gilliam, in a C57BL/6 murine model. Using equivalent infectious doses, we assessed splenic B cell and germinal center (GC) responses during acute infection through flow cytometry, immunohistology, serological analyses, and RNA sequencing.

**Results:**

Karp infection resulted in high tissue bacterium burdens and 50% mortality rates, whereas Gilliam infection was self-healing with limited bacterium dissemination and growth. Yet, Gilliam induced strong splenic B cell responses, as judged by total numbers of B cells, follicular B cells, and marginal zone B cells, which correlated with serum IgG and IgM levels. Given that Karp- but not Gilliam-infected spleens displayed GC disorganization/loss and MZ abrogation, we compared splenic RNAseq profiles. On day 4 post-infection, Karp induced significant elevation of transcriptional inflammatory signatures (*Ccl2*, *Il33*, *Ifng*) and inflammatory gene pathways (*Il1*, *Il6*, *Tnf*, *Ifng*), the characteristics of severe scrub typhus. Moreover, IPA analysis revealed Karp-induced neutrophil activation/degranulation and defense response pathways, which were in sharp contrast to Gilliam-induced upregulation of phagocytosis signaling pathways. Splenocyte flow cytometry confirmed significantly higher influx of activated phagocyte subsets (neutrophils, M1 macrophages, and inflammatory monocytes) during Karp infection than Gilliam infection, indicating greater infiltration of innate immune cells in severe scrub typhus.

**Discussion:**

Collectively, this study provides the first lines of evidence for Ot strain-related, cellular and humoral immune signatures in the spleens, which help understand differential host immune responses during acute scrub typhus.

## Introduction

*Orientia tsutsugamushi* (*Ot*) is an obligately intracellular bacterium and causative agent of the understudied and underdiagnosed febrile illness, scrub typhus. Within endemic regions, commonly known as the tsutsugamushi triangle, there are an estimated one million cases annually, although the actual number of cases is likely much higher than this estimation due to lack of surveillance, rural prevalence, and difficulties with diagnosis (1). Scrub typhus can be life-threatening, particularly in cases with delayed or inadequate antibiotic treatment, where median case fatality rates increase from 1.4% with treatment to 6% without treatment (2–4). In severe scrub typhus cases, multiple organs can become infected and manifestations such as acute respiratory distress syndrome, acute renal failure, meningoencephalitis, disseminated intravascular coagulation, or septic shock can occur (5). Although historically confined to endemic regions, the recent reports of new *Orientia candidatus* species in Chile (*O. chiloensis*) and Dubai (*O. chuto*), along with the detection of *Ot* in mite vectors in North Carolina, have drawn renewed attention to this neglected yet life-threatening tropical disease (6–9). At least 20 antigenically distinct *Ot* strains have been reported (10), with different epidemiological prevalence and geographic distributions. For example, clinical isolates related to Karp, Gilliam, and Kato prototypes are found throughout the tsutsugamushi triangle, especially in East Asian countries (10, 11). Importantly, Karp and Gilliam are clinically prevalent strains, representing approximately 65% and 26% of global cases, respectively (12). Karp-infected patients had greater disease severity than Gilliam-infected cases, based on clinical parameters (13) and blood bacterial burdens (14).

Scrub typhus immunity seems to be short-lived, waning in a few months or years post-infection (15–17). For example, Ha et al. reported that patients’ CD4^+^ and CD8^+^ T cells specific to the *Ot* major outer membrane protein, TSA56, declined one year after infection and were almost undetectable by two years post-infection (16). Likewise, humoral immunity declined quickly, as 50% of scrub typhus cases became sero-negative by 49 weeks post-infection (15), while some cases nearly or totally lost *Ot* antigen-specific antibodies around two years (16). To date, no clinical studies have addressed possible mechanisms for this nondurable immunity, although serum analyzes indicate type 1-skewed inflammatory cytokine/chemokine profiles (high IFNγ, TNFα, IL-6, IL-8, IL-12p40, MCP-1, MIP-1β, CXCL10) at acute infection stages (18–21), as well as a positive correlation between high TNFα, IL-8, and IL-10 levels and scrub typhus severity (19–21).

Experimental murine models are valuable tools for characterization of *Ot* strain virulence, disease pathogenesis, and host immune landscapes. For example, through comparative studies with different mouse models (inbred, outbred, gene-targeted knockouts) and different *Ot* strains, we and others have shown Karp as high virulence and Gilliam as intermediate virulence, respectively (22–25). For comparable inoculation doses in outbred CD-1 mice and IFNγ pathway-deficient C57BL/6 mice, Karp led to 100% lethality, whereas Gilliam caused approximately 50% lethality, mostly due to differences in bacterium growth/killing, host cell injury, innate and adaptive immune responses (23, 25). High mortality rates in Karp-infected mice were likely due to uncontrolled bacterium replication and severe lung pathology, accompanied with extensive influx of activated immune cell subsets (M1 macrophages, neutrophils, NK cells, and CD4^+^) at early stages and prior to host death (23).

While we begin to understand differential cellular immune profiles during severe vs. mild scrub typhus, very little is known about their corresponding B cell and GC responses (26). Since GC responses are critical to the development of humoral immunity after infection (27), it is not surprising for other human pathogens to evolve strategies to subvert humoral immune responses or disrupt splenic microarchitecture (28, 29). We speculated that severe vs. mild scrub typhus may have distinct splenic immune landmarks at early stages of infection.

In this study, we used our established C57BL/6 models and same viable doses of Karp and Gilliam strains for comparative studies of humoral responses. We found that severe Karp infection, but not self-healing Gilliam infection, resulted in GC collapse and splenic MZ abrogation, as judged by immunohistology and confocal microscopy. At days 4 and 8 post-infection, Karp-associated transcriptomics were characterized with elevated inflammation genes (*Ccl2*, *Il6*, *Il10*, *Ifng*), TNF/IFNγ/IL-1/IL-17-related pathways, and monocyte/macrophage/neutrophil influx. Flow cytometric analyses confirmed a Karp-associated increase of influx and activation of myeloid cell subsets, whereas Gilliam infection mounted balanced immune responses. This is the first study to evaluate differential immune responses to two prevalent *Ot* strains in the context of the splenic immunological niche, revealing new insights into the collapse of GCs and the virulence-associated alterations in host immune responses.

## Materials and methods

### Mouse infection and ethics statement

Female C57BL/6 mice (#000664, Jackson Laboratory) were maintained under specific pathogen-free conditions. Animals used were at 8–12 weeks of age, following protocols approved by the Institutional Animal Care and Use Committee (IACUC#1902006 and #2101001A) at the University of Texas Medical Branch (UTMB) in Galveston, TX. Mouse infection studies were conducted in the Galveston National Laboratory ABSL3 facilities using procedures approved by the Institutional Biosafety Committee, in accordance with Guidelines for Biosafety in Microbiological and Biomedical Laboratories. UTMB complies with the USDA Animal Welfare Act (Public Law 89-544), the Health Research Extension Act of 1985 (Public Law 99-158), the Public Health Service Policy on Humane Care and Use of Laboratory Animals, and the NAS Guide for the Care and Use of Laboratory Animals (ISBN-13). UTMB is registered as a Research Facility under the Animal Welfare Act and has current assurance on file with the Office of Laboratory Animal Welfare, in compliance with NIH policy. Both *Ot* Karp and Gilliam stocks were prepared in L929 cells, and the inoculum infectivity titer of prepared stocks was determined, as in our previous report (30). Mice were intravenously inoculated with a viable dose (6.8 × 10^4^ FFU, 200 µl) of Karp, Gilliam, or PBS (mock) and monitored daily for weight loss, signs of disease, and survival. Disease scores were assigned to mice daily, ranging from 0-5, based on an institute approved protocol for animal sickness, as in our previous reports (24, 31). At indicated times, serum and spleen samples (4-5/group) were collected and prepared for subsequent immunological analyses. All independent studies were performed with the same batch of bacterial stocks. Data shown are representative of two independent experiments.

### Flow cytometry

Spleens were passed through 70-μm cell strainers in RPMI 1640 medium to prepare single-cell suspensions which were then treated with Red Blood Cell Lysis Buffer (Sigma-Aldrich). Cells were first blocked with FcγR blocker (BioLegend), followed by staining with Fixable Viability Dye eFluor 780 (eBioscience) or LIVE/DEAD Fixable Blue Dead Cell Stain (Thermo Fisher Scientific) and fluorochrome-labeled antibodies (Abs). The Abs below were purchased from either BD Biosciences, BioLegend, Invitrogen, or Tonbo Biosciences for B and T cell staining: BV785-anti-B220, V450-anti-CD3, APC-anti-CD138, PerCPCy5.5-anti-CD38, Alexa fluor488-anti-GL7, PE-Dazzle594-anti-CD23, PE-Cy7-anti-CD21/35, PE-anti-IgM, BV510-anti-IgD, PE-Cy7-anti-CD3, PerCP-Cy5.5-anti-CD4, FITC-anti-CD8, BV711-anti-CD44, BV605-anti-PD-1, BV421-anti-CXCR5, and PE-anti-FOXP3. For myeloid cell staining, the following Abs were purchased from BD Biosciences, BioLegend, and eBioscience: FITC-anti-CD11b, APC-anti-Ly6G, PE-Dazzle-594-anti-Ly6C, RB545-anti-CD80, APC-Cy7-anti-CD63, BV421-anti-F4/80, RB780-anti-SCA-1, and BV650-anti-CCR2. Cells were fixed in 2% paraformaldehyde overnight at 4°C prior to data acquisition. Data was acquired on a BD FACSymphony A5 in the UTMB Flow Cytometry Core and analyzed by using FlowJo software version 10.7.2 (BD Bioscience).

### Immunohistology

Spleen tissues were fixed in 4% paraformaldehyde/5% sucrose/PBS overnight. Tissues were transferred into 20% sucrose/PBS for 24 h at 4 °C, followed by 30% sucrose/PBS for another 24 h at 4 °C. Spleens were embedded in O.C.T. compound (Sakura Finetek). Frozen cryosections (7-μm) were blocked with 1% BSA/0.3 M glycine/PBS for 30 min. They were then incubated with rat IgG2a anti-B220 (1:175), rat IgM anti-GL-7 (1:175), biotin anti-CD3 (1:200) Abs for 1 h at room temperature (BioLegend, clones RA3-6B2, GL-7, 17A2, respectively). Cryosections were then stained with secondary Abs, Alexa Fluor 594-conjugated mouse anti-rat IgG2a (1:200, clone MRG2a-83, BioLegend), Alexa Fluor 488-conjugated goat anti-rat IgM (1:200, clone A21212, Invitrogen), streptavidin cyanine 5 (1:200, BioLegend) for 1 h. Staining with primary or secondary antibodies alone served as negative controls. At least 4–5 fields of each spleen section were imaged at the UTMB Optical Microscopy Core on a Zeiss LSM 880 confocal microscope (Carl Zeiss Microscopy LLC, equipped with ApoTome and Zen imaging software). The 488, 561, and 633 excitation lasers under 63x oil immersion objective were used. Acquisition settings were identical among samples of different experimental groups, and representative images are presented from each time point.

### Quantitative reverse transcription PCR

Splenic tissues were incubated in RNA*Later* (Qiagen) at 4°C overnight for inactivation. Tissues were homogenized in a BeadBlaster 24 Microtube Homogenizer (Benchmark Scientific) with RLT lysis buffer (Qiagen) and metal beads. The RNEasy Mini kit (Qiagen) was used for total RNA extraction i Kit (Qiagen). The iScript Reverse Transcription kit (Bio-Rad) was used for cDNA synthesis and amplification in a 10 μL-reaction mixture containing 5 μL of iTaq SYBR Green Supermix (Bio-Rad) and 0.5 μM each of gene-specific forward and reverse primers. qRT-PCR assays were performed on a CFX96 Touch Real-Time PCR Detection System (Bio-Rad), and PCR assays were denatured for 30s at 95 °C, followed by 40 cycles of 15s at 95 °C, and 60s at 60 °C. Melt curve analysis was performed to check specificity of amplification. Relative quantitation of mRNA expression was calculated utilizing the 2^-ΔΔCT^ method. Primers used in qRT-PCR analysis are listed in **Supplementary Table S1**.

### Quantitative PCR for tissue bacterial burden

DNA extraction was performed by using a DNeasy Blood & Tissue Kit (Qiagen) followed by qPCR assay, as in our previous report (32). The copy number for the 47-kDa gene was determined by known concentrations of a control plasmid containing single-copy insert of the gene. Gene copy numbers were determined via 10-fold serial dilution of the *Ot* 47-kDa plasmid. Bacterial burdens were normalized to total nanogram (ng) of DNA per µL. Data are expressed as the gene copy number of 47-kDa gene per ng of DNA.

### Enzyme-linked immunosorbent assay

Serum was separated from whole blood samples in blood separation tubes (BD Bioscience) by centrifuging at 9,000 g for 2 min. For analysis of antigen-specific Ab responses, we used our reported procedures (26), with minor modifications of coat proteins. Briefly, 96-well plates were coated overnight with recombinant Karp TSA56 and Gilliam TSA56 proteins that were generated by Genscript (2 μg/mL each, mixed at a 1:1 ratio in PBS) and then blocked with 0.5% BSA. Serum samples were serially diluted at 1:3 until ELISA endpoint titers were determined. Detection was performed utilizing the following horseradish peroxidase-conjugated primary antibodies that were diluted 1:3,000 in a blocking buffer: goat anti-mouse IgM and anti-mouse IgG (Southern Biotech). Visualizing reagent utilized was the 1-Step Ultra TMB ELISA Substrate Solution (Thermo Fisher Scientific). Optical density was measured on the BioTek Epoch microplate spectrophotometer. Area under the curve (AUC) analysis was performed on each curve (5 per group) at every time point, as reported (33).

### Splenic RNAseq

Splenic tissues were incubated in RNA*Later* (Qiagen) at 4°C overnight, followed by RNA extraction utilizing the RNeasy Mini Kit (Qiagen). RNAseq analysis was performed by Novogene (San Jose, CA). Following quality control assessment, mRNA was purified from total RNA with poly-T oligo-attached magnetic beads. Fragmentation, cDNA synthesis and library preparation were then performed by Novogene. The Illumina Novaseq Platform NovaSeq X Plus was used for sequencing. The *Mus musculus* genome build mm10 was used as a reference genome. Raw data were first evaluated for quality control to exclude reads with adapter, poly-N, and low quality, and thus only clean data were further examined. Normalization and analysis were completed by Novogene using featureCounts v1.5.0-p3 for quantification of gene expression levels, DESeq2 R package and for differential expression analysis, and clusterProfiler R package for Gene Ontology, KEGG, Reactome, Wikipathways, and Pathway Interaction DB databases enrichment analysis of differentially expressed genes. Ingenuity Pathway Analysis (IPA) was performed for Karp-vs- Gilliam comparison at each time point to identify canonical pathways with results filtered by a Benjamini-Hochberg corrected *p*-value. Results were reported with z-scores and filtered by an adjusted *p*-value < 0.05. All RNAseq data discussed in this publication have been deposited in NCBI’s Gene Expression Omnibus and are accessible through GEO Series accession number GSE290855 (https://www.ncbi.nlm.nih.gov/geo/query/acc.cgi?acc=GSE290855).

### Statistical analysis

All data (except for RNAseq data) were analyzed by using GraphPad Prism software. Data were presented as mean ± standard deviation (SD) or standard error of mean (SEM). Bodyweight change data were statistically analyzed with two-way ANOVA and Šídák’s multiple comparisons test. Survival data were assessed using a survival curve comparison and Log-rank (Mantel-Cox) test, log-rank test for trend, and Gehan-Breslow-Wilcoxon test. Disease score, splenic bacterial burden qPCR data, ELISA AUC, qRT-PCR, and flow cytometry data were analyzed with one-way ANOVA and Tukey’s multiple comparisons test *Post Hoc* for comparisons between groups. Statistically significant values are denoted as *p < 0.05, *** *p* < 0.001, and **** *p* < 0.0001, respectively, or ns, for no significance.

## Results

### Distinct disease outcomes induced by *Ot* Karp vs. Gilliam strain

**Figure 1:**
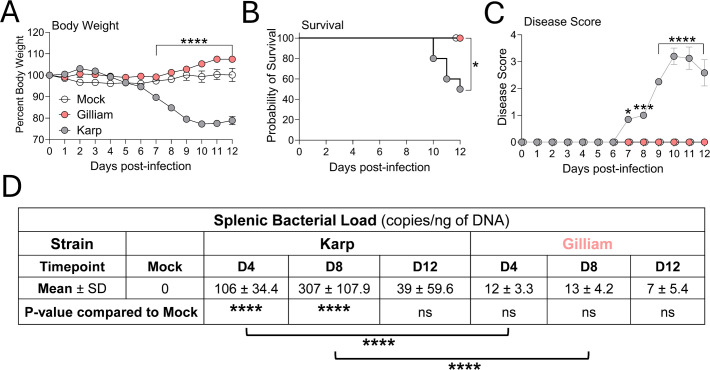
Disease progression kinetics in *Ot* Karp- or Gilliam-infected mice. C57BL/6 mice (5 per group) were intravenously infected with Karp or Gilliam strain (6.8 × 10^4^ FFU) or PBS (mock controls) and monitored daily for **(A)** body weight, **(B)** survival rates, and **(C)** disease scores. Mice were euthanized at D0 (mock), D4, D8, and D12 for spleen and serum samples harvest and analyses. **(D)** Splenic bacterial burdens were measured by qPCR. Results are shown as mean ± SD from a single experiment and are representative of two independent experiments with similar trends. For statistical analysis, two-way ANOVA was used with a Šídák’s multiple comparisons test **(A)**. Survival curves were analyzed by survival curve comparison and Log-rank (Mantel-Cox) test, log-rank test for trend, and Gehan-Breslow-Wilcoxon test **(B)**. Disease score and splenic bacterial load data were analyzed by one-way ANOVA and Tukey’s multiple comparisons test **(C, D)**. Asterisks or ns were representative of comparison between Karp- and Gilliam-infected mice at each timepoint, unless otherwise stated. ns, no significance; *p < 0.05; ***p < 0.001; ****p < 0.0001.

Karp and Gilliam strains account for most of human scrub typhus cases; yet no reports are available for their splenic immune responses. Here, we infected C57BL/6 mice with an equivalent viable dose of Karp or Gilliam and measured kinetics of disease progression daily. As shown in **Figure 1**, Karp infection led to progressive weight loss (beginning at D4 and reaching >20% body weight loss at D10), with the highest disease score at D10 and 50% lethality at D12. In contrast, Gilliam-infected mice showed no weight loss, disease score, or mortality. At D4 and D8, Karp-infected spleens contained significantly higher bacterial burdens than those in Gilliam infection (**Figure 1D**), resulting in a 15-fold difference between the two groups at the peak of infection (D8). Therefore, for the inoculation dose we used, Karp caused a severe and sublethal infection, while Gilliam did not cause any disease signs. Our findings support the distinct clinical outcomes for these two *Ot* strains in multiple mouse models established in our laboratory (23–25, 34).

### Antigen-specific IgM and IgG levels during Karp vs. Gilliam infection

**Figure 2:**
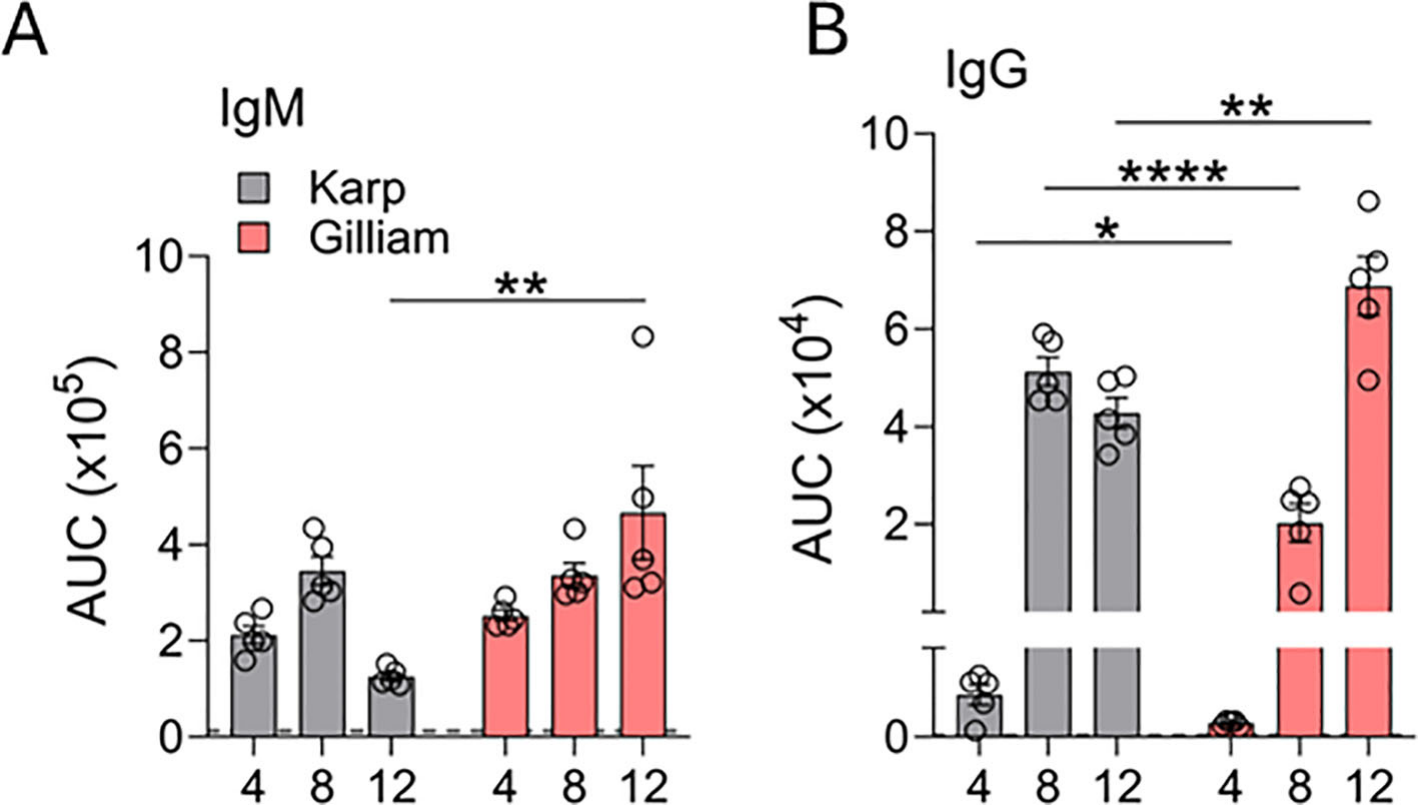
Differential serum IgM and IgG responses in Karp- vs. Gilliam-infected mice. Mice were infected, as described in **Figure 1**. Infected and mock serum samples (5 per group) were serially diluted at 1:3 ratios. Antibodies specific to TSA56 (both Karp and Gilliam recombinant proteins, 1:1 mixed) were measured via indirect ELISA assays for IgM and IgG responses. AUC analyses of ELISA antibody curves were performed on **(A)** IgM and **(B)** IgG data; AUC values shown in bar plots were calculated from OD_450_ measurements across the full dilution ranges. Data are shown as mean ± SEM from a single experiment and are representative of two independent experiments with similar trends. For statistical analysis, one-way ANOVA was used with a Tukey’s multiple comparisons test for comparison of Karp- and Gilliam-infected samples at each timepoint Asterisks were representative of comparison between Karp- and Gilliam-infected mice at each timepoint. *p < 0.05; **p < 0.01; ****p < 0.0001.

For all ELISA assays, Karp and Gilliam TSA56 recombinant proteins were mixed at a 1:1 ratio and used as coating antigens to allow unbiased comparison of antigen-specific antibody responses between mice infected with Karp or Gilliam strains. We measured serum total IgM (**S1 Fig**) and IgG titers (**S1 Fig**) via indirect ELISA and then performed area-under-the-curve (AUC) analyses, as in our previous report (26). In comparison to mocks, both Karp- and Gilliam-infected mice had elevated and comparable IgM levels at D4 and D8 (**Figure 2A**). At D12, however, Karp-infected mice demonstrated a drop in IgM levels, and these mice had significantly lower (~3.7-fold) IgM levels than Gilliam-infected mice (**Figure 2A**). In terms of IgG titers, D4 and D8 of Karp infection respectively stimulated 3- and 2.5-fold higher responses than those of Gilliam infection (**Figure 2B**). By D12, however, Gilliam infection induced significantly greater (1.6-fold higher) IgG responses than Karp infection. Dynamics of IgG levels differed between Karp and Gilliam infection, with IgG reaching a peak at D8 for Karp, while IgG levels peaked at D12 in Gilliam infection. Collectively, these results demonstrate that Karp and Gilliam infections generate distinct temporal patterns of IgM and IgG production, reflecting clear strain-dependent differences in antibody responses.

### Splenic GC disorganization during Karp vs. Gilliam infection

**Figure 3:**
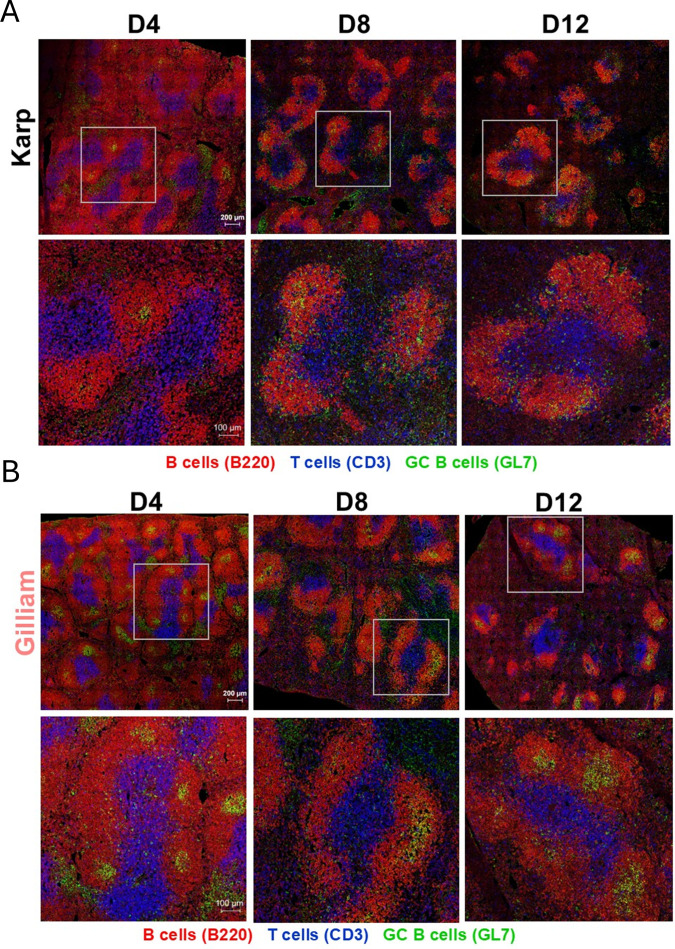
GC disorganization occurs in *Ot* Karp, but not Gilliam, infection. Mice were infected, as described in **Figure 1**. Spleen cryosections were prepared and co-stained for B cells (B220), T cells (CD3), and GC B cells (GL7). Images were captured by using confocal microscopy (Zeiss LSM 880). Shown are splenic white pulp overview (scale bar, 200 μm) and high-magnification images (scale bar, 100 μm) from **(A)** Karp and **(B)** Gilliam infection, respectively.

Splenic architecture and GC organization are important indicators for humoral immunity and host defense (29). To better understand clinical outcomes and splenic responses associated with these two *Ot* strains, we performed immunostaining for B220^+^ B cells (red), CD3^+^ T cells (blue), and GL7^+^ GC B cells (green), respectively. As shown in (**Figures 3A, B**), overview scans revealed that both infection groups had comparable and well-organized white pulp regions with expanded T cell zones and discernable GCs within B cell follicles at D4. As infection progressed, both *Ot* strains showed reduced T cell zone sizes, increased detection of T cells outside of white pulp regions, and greater space between white pulp regions, but Karp-infected mice had less defined T cell zones and B cell follicles. We then assessed GC formation during infection by capturing images of individual white pulp regions (**Figures 3A, B**). While both infection groups had identifiable GCs in B cell follicles at D4, Karp-infected mice display scattering of GC B cells within B cell follicles and less apparent GC structures at D8. While Gilliam-infected spleens maintained clear GCs in B cell follicles at D12, no identifiable GCs were detected in Karp-infected mice. Also, while we found shrunken white pulp regions in spleens of Karp- and Gilliam-infected groups, only Karp infection induced GC disorganization, demonstrating GC collapse and GC B cell scattering as unique characteristics. Overall, our findings suggest *Ot*-associated and strain-associated splenic architectural trends.

### Selective impairments of T- and B-cell subsets during Karp, but not Gilliam, infection

**Figure 4:**
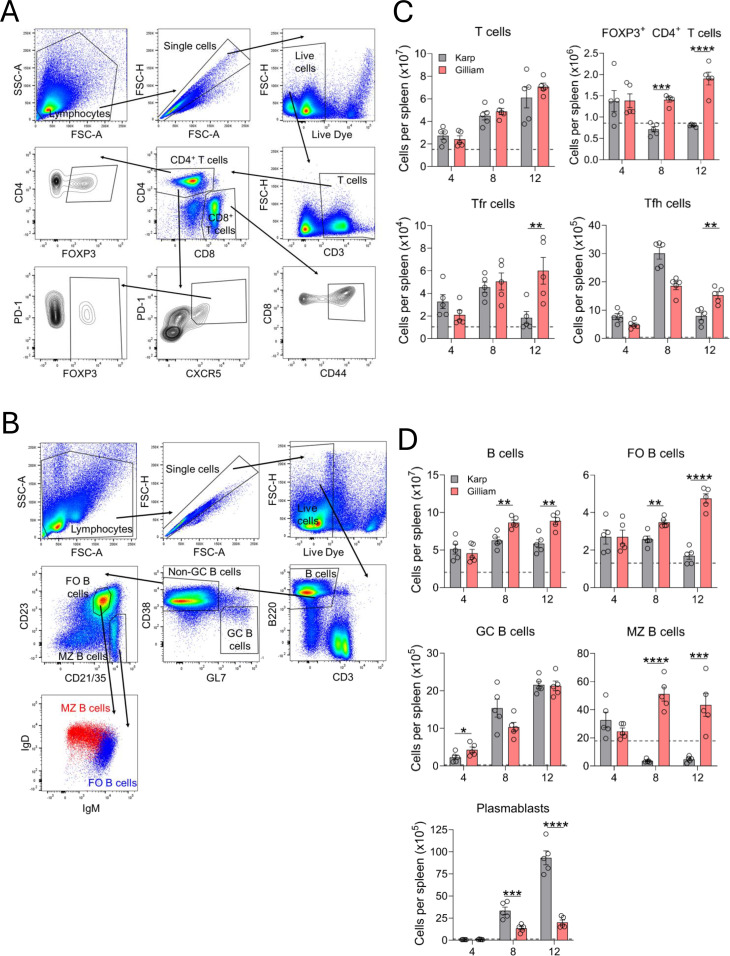
Gilliam infection induced more robust humoral immune responses as compared to Karp infection. Mouse spleens were harvested at given time points (5 per group), as described in **Figure 1**. Single-cell suspensions were prepared and stained for indicated cell surface markers. Gating strategies are shown for **(A)** T cell subsets and **(B)** B cell subsets, respectively. Flow cytometric results are presented as absolute numbers of various **(C)** T cell populations and **(D)** B cell populations. Results from mock groups are presented as dashed lines. Data are shown as mean ± SEM from a single experiment and are representative of two independent experiments with similar trends. For statistical analysis, one-way ANOVA was used with a Tukey’s multiple comparisons test for comparison of Karp- and Gilliam-infected samples at each timepoint. Asterisks were representative of comparison between Karp- and Gilliam-infected mice at each timepoint. *p < 0.05; **p < 0.01; ***p < 0.001; ****p < 0.0001.

Since the loss and/or re-distribution of various cellular components can contribute to splenic GC collapse (35, 36), we used multi-color flow cytometry to assess key T- and B-cell subsets during infection (**Figures 4A, B**). The total numbers of CD3^+^, CD4^+^ and CD8^+^ T cells, as well as CD44^+^ antigen-experienced CD8^+^ T cells were comparable between two infection groups at various timepoints; all of them were significantly higher than mock groups (dashed lines, **Figure 4C**; **Supplementary Figure S2**). Compared with Karp counterparts, Gilliam-infected mice had significantly higher total numbers of CD4^+^FoxP3^+^ regulatory T cells at D8 and ~2.4-fold higher at D12, as well as 3.2-fold higher CD4^+^CXCR5^hi^PD-1^hi^FOXP3^+^ T follicular regulatory cells (Tfr) at D12, respectively. For Karp infection, T follicular helper (Tfh) cells (CD4^+^CXCR5^hi^PD-1^hi^FOXP3^-^) peaked at D8, but absolute numbers fell sharply by 3.8-fold at D12, correlating with the loss of GCs (**Figure 3**).

During Gilliam infection, there was a steady increase in the absolute numbers of total B220^+^ B, CD38^+^CD23^+^ follicular (FO) B cells, CD38^-^GL7^+^ GC B cells, and IgD^+^CD23^+^ marginal zone (MZ) B cell as compared to the mocks (dashed lines, **Figures 4B, D**). In contrast, Karp infection only showed an expansion in GC B cells throughout infection, while MZ B cell numbers dropped after D4. Given these opposite trends, Gilliam-infected mice had nearly 3-fold higher FO B cell numbers at D12, as well as 14-fold and 9-fold MZ B cell numbers at D8 and D12, than Karp-infected counterparts. Notably, although Karp- and Gilliam-infected mice exhibited similar GC B cell numbers at D12, this timepoint coincided with pronounced GC disorganization in Karp-infected mice (**Figure 3**), suggesting that GC B cell abundance alone may not reflect proper GC structure or function. The marked depletion of marginal zone B cells during Karp infection may further contribute to splenic architectural disruption, as these cells play important roles in maintaining splenic organization and facilitating antigen capture and delivery to follicles. Plasmablasts (B220^+^ CD138^+^) were significantly higher in absolute cell number at D8 and D12 during Karp infection, than Gilliam infection, suggesting that Karp strain induced stronger extrafollicular responses. Altogether, these findings indicate that Gilliam infection induced coordinated B cell responses, characterized by sustained Tfh/Tfr populations and preservation of FO and MZ B cell compartments, consistent with the maintenance of organized GC structures. In contrast, Karp infection resulted in disrupted B and T cell responses, including reduced CD4^+^FoxP3^+^ regulatory T cells at D8 and D12, marked decline in Tfh and Tfr populations, and depletion of MZ B cells at later timepoints, which may correlate with the GC disorganization observed histologically.

### Disrupted splenic marginal zones (MZ) during Karp infection

**Figure 5:**
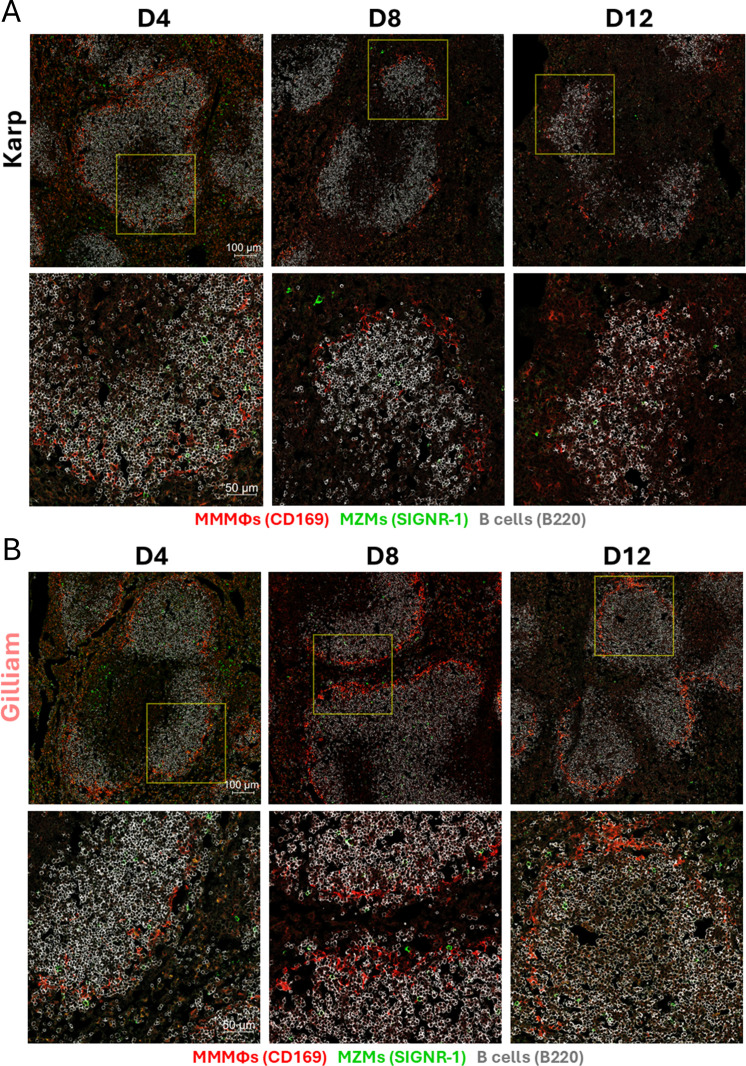
Abrogation of marginal zone occurs exclusively in Karp-infected mice. Mice were infected, as described in **Figure 1**. Frozen cryosections of spleen were co-stained for B cells (B220), MZ macrophages (SIGNR-1), and metallophilic macrophages (CD169). Images were captured by using confocal microscopy (Zeiss LSM 880). Shown are overviews (scale bar, 100 μm) and high-magnification images (scale bar, 50 μm) from **(A)** Karp and **(B)** Gilliam infection, respectively.

Having demonstrated Karp infection in the spleen (**Figure 1**) and severe reduction of MZ B cell numbers as disease progress (**Figure 4D**), we then investigated the condition and organization of the splenic MZ, especially for GC-related macrophage subsets, as macrophages are key host cells for *Ot* replication (37–39). We stained cryosections for B cells (B220), MZ macrophages (SIGNR-1), and metallophilic macrophages (CD169) and generated images via confocal microscopy (**Figures 5 A, B**). At D4 of both infection groups, the MZs formed a continuous outer layer around B cell follicles with strong, detectable signals for CD169^+^ and SIGNR-1^+^ cells. MZ alterations were noted in Karp-infected spleens at D8, as judged by reduced detection of MZ and metallophilic macrophages, while Gilliam-infected spleens maintained positive signal for CD169^+^ metallophilic macrophages, which formed a dense layer around the white pulp. In sharp contrast to Gilliam samples at D12, Karp-infected spleens had relatively weak staining for MZ and metallophilic macrophages or completely lost positive staining in some MZ areas. Together, these results highlight significant differences in the splenic microarchitecture between Karp- vs. Gilliam-infected mice. Given the role of splenic MZs in aiding GC responses and orchestrating cytokine and cellular immune responses, the depletion of this lymphoid microstructure in Karp infection may impair not only humoral immune responses, but also cellular immune responses, as suggested in other infectious disease models (40–43).

### Enhanced transcriptional signatures of inflammatory and leukocyte recruitment pathways during Karp infection

**Figure 6:**
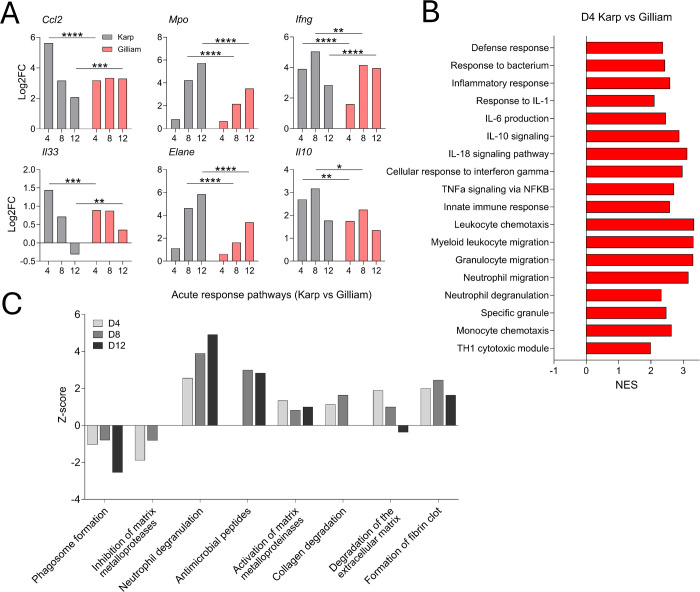
Transcriptomic analysis of splenic environment in Karp- vs. Gilliam-infected mice. Mice were infected, and spleen tissues were collected at indicated time-points, as described in **Figure 1**. RNAseq was performed (4 per group) and analyzed by Novogene. Differentially expressed gene comparisons (D4 Karp vs Mock, D4 Gilliam vs Mock, D8 Karp vs Mock, D8 Gilliam vs Mock, D12 Karp vs Mock, and D12 Gilliam vs Mock) were identified. **(A)** Select genes are plotted using log_2_-fold change and displayed. **(B)** Differentially expressed genes relating to inflammatory and innate immune cell pathways were identified by gene set enrichment analysis from D4 Karp vs Gilliam comparison. Pathways upregulated in Karp infection appear as positive NES values. **(C)** IPA analysis of pathways involved in acute phase response from Karp versus Gilliam comparisons at D4, D8, and D12. Results were filtered by a Benjamini-Hochberg corrected *p*-value. Pathways upregulated in Karp infection show positive z-scores, while pathways upregulated in Gilliam infection show negative z-scores. *p < 0.05; **p < 0.01; ***p < 0.001; ****p < 0.0001.

To further define possible mechanisms underlying Karp-associated GC loss (**Figures 4**, **5**), we performed RNAseq analyses (4 samples/group). Principal component analysis confirmed that Karp and Gilliam samples clustered distinctively at given timepoints (**S3A Fig**). Comparison of Karp- vs. Gilliam-infected spleens at D4, D8, and D12 revealed 454, 843, and 1,121 differentially expressed genes, respectively (**S3B Fig**). To evaluate potential genes encoding biomarkers associated with scrub typhus and inflammation, we plotted log_2_-fold change of various differentially expressed genes within each group relative to the mocks (**Figure 6A**). Consistent with our previous lung-focused reports (23, 44), Karp infection induced significantly higher expression levels of *Ccl2* (1.8-fold) and *Il33* (1.6-fold) at D4, as well as higher *Ifng* (2.4-fold) and *Il10* (1.4-fold) at D8, than Gilliam infection. Additional inflammation-related differentially expressed genes between Karp versus Gilliam also showed elevated expression of inflammatory cytokine and chemokine genes at D4 in Karp-infected mice compared to Gilliam-infected mice (**S6 Fig**). These tissue-based findings were relevant as both IFNγ and IL-10 are linked to severe scrub typhus in patients (18). We then performed gene-set-enrichment analysis for *Ot* strain comparison, especially for genes involved with defense and inflammatory responses (**Figure 6B**). Normalized enrichment scores revealed significant upregulation of defense response pathways involved in “Response to bacterium” in Karp infection at D4. We also identified the upregulation of gene sets associated with inflammatory response pathways during Karp infection, including signaling pathways related to proinflammatory cytokines (*Il1*, *Il6*, *Il18*, *Ifng*, *Tnf*), as well as the anti-inflammatory, severe scrub typhus-related cytokine *Il10*. Our analysis also uncovered increased expression of leukocyte recruitment pathways, including granulocytes and monocytes. Together, our results reveal a distinctive transcriptional profile in Karp-infected spleens characterized by upregulation of proinflammatory cytokine and leukocyte recruitment–associated gene signatures.

To further characterize the acute response pathways during infection, we used IPA analysis to visualize the expression of pathways from Karp vs. Gilliam comparisons at all examined time points (**Figure 6C**). Gilliam infection showed upregulation of phagosome formation pathway compared to Karp counterparts during infection, possibly demonstrating increased immune recognition and subsequent phagocytosis. Notably, we found significant upregulation of neutrophil degranulation pathway at all time points in Karp infection, which corresponded with the increased expression of pathways capable of extracellular matrix (ECM) remodeling, including activation of matrix metalloproteinases (MMP), collagen degradation, and degradation of the ECM. Of note, principal component analysis identified that while *Mpo* and *Elane* (neutrophil degranulation-related genes) reached peak at D12 in both infection groups, their expression levels were 1.6- and 1.7-fold higher in Karp than in Gilliam, respectively. In contrast, Gilliam-infected spleens upregulated the inhibition of matrix metalloproteases pathway at D4 and D8, which could display resistance of ECM degradation in Gilliam infection. These RNAseq findings were consistent with qRT-PCR–measured expression of *Mmp8* and *Mmp9*, supporting increased transcriptional activity of ECM remodeling-associated genes during Karp infection, which showed significantly higher expression in Karp-infected spleens compared to Gilliam counterparts (**Supplementary Figure S5**).

Next, we assessed the expression of adaptive immune-related pathways between two *Ot* strains by utilizing IPA analysis at all time points (**Supplementary Figure S4A**). Karp infection showed marked upregulation of CTLA4 signaling in cytotoxic T lymphocytes, particularly at D12. In contrast, Gilliam-infected spleens showed upregulation of lymphoid and non-lymphoid immunoregulatory interactions pathways at all time points, as well as elevated expression of Th1 and Th2 pathways at D12. Utilizing B cell activation gene lists derived from GO and Biological Process, we identified 33 differentially expressed genes relevant to B cell activation (*Pax5*, *Cd19*, *Aicda*, *Cd40*, etc.) at D12 (**Supplementary Figure S4B**). Among these differential genes, 28/33 genes (85%) were upregulated in Gilliam infection, while the remaining 5 differentially expressed genes were upregulated in Karp infection, implying attenuated B cell activation during Karp infection. Collectively, these transcriptional profiles reveal significant differences in B- and T-cell responses between Karp and Gilliam infections, highlighting the deficiency in adaptive immune signaling pathways in Karp-infected spleens.

## Significant activation of myeloid cell subsets in Karp-infected spleens

**Figure 7:**
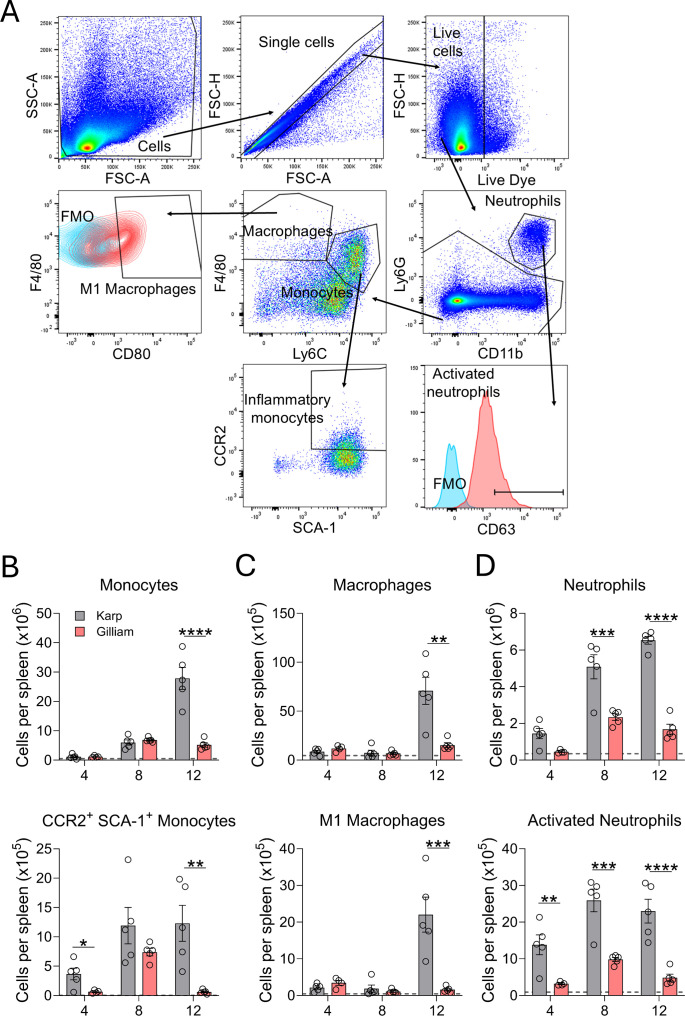
Karp infection induces significant influx of myeloid cell subsets. Mock and infected mice were euthanized, and their spleens were harvested at given time points (5 per group), as described in **Figure 1**. Single-cell suspensions were prepared and stained for indicated cell surface markers. **(A)** Gating strategy for myeloid cell subsets is shown. Absolute cell numbers of **(B)** monocytes and inflammatory monocytes, **(C)** macrophages and M1 macrophages, and **(D)** neutrophils and activated neutrophils are displayed. The absolute cell numbers of the mock group are represented by the dashed grey line. Data are shown as mean ± SEM from a single experiment and are representative of two independent experiments with similar trends. For statistical analysis, one-way ANOVA was used with a Tukey’s multiple comparisons test for comparison of Karp- and Gilliam-infected samples at each timepoint. Asterisks were representative of comparison between Karp- and Gilliam-infected mice at each timepoint. *p < 0.05; **p < 0.01; ***p < 0.001; ****p < 0.0001.

Given that CCR2^+^SCA-1^+^ monocytes are linked to GC disruption during *Salmonella* infection (45), we used multi-color flow cytometry to identify several myeloid cell subtypes (**Figure 7A**). As shown in **Figure 7B**, Karp infection led to significantly higher numbers of F4/80^+^Ly6C^hi^ monocytes at D12, as well as CCR2^+^SCA-1^+^ monocytes at D4 and D12, than Gilliam infection. Notably, CCR2^+^SCA-1^+^ monocytes in Gilliam-infected spleens exhibited a transient increase at D8 that returned to baseline levels by D12, whereas the frequency of CCR2^+^SCA-1^+^ monocytes in Karp-infected spleens was approximately 20-fold higher (12.3% vs 0.6%) at D12. Gilliam infection also showed minimal influx of F4/80^hi^Ly6C^lo^ macrophages and CD80^+^ M1 macrophages, whereas the recruitment of these cell subsets was robust at D12 (**Figure 7C**). Given our finding of several upregulated pathways involved in neutrophil migration and degranulation in Karp infection (**Figure 6**), we assessed the number of neutrophils and activated neutrophils. As shown in **Figure 7D**, Karp infection led to a rapid and significant increase of Ly6G^hi^CD11b^hi^ neutrophils at D8 and D12. A significant and differential increase of activated CD63^+^ neutrophils was detected even at D4; such trends were maintained at D8 and D12, respectively. Neutrophil-related responses were minor or modest during Gilliam infection. Collectively, our flow cytometry results reveal *Ot* strain-dependent infiltration and activation of myeloid cell subsets, which support and are consistent with our immunohistology and RNA-seq findings.

## Discussion

Reported studies from murine scrub typhus models have clearly indicated that severe Karp infection is characterized by strong inflammatory transcriptional signatures and Th1/M1 macrophage-skewed reactions in all major organs (23, 25, 46), as well as disorganized splenic architectures and GC formation (26). However, immune cell- or organ-based mechanistic studies between or among clinical prevalent *Ot* strains remain very limited (23, 25, 47). In this study, we used the same inoculation dose of *Ot* Karp (highly virulent strain) and Gilliam (relatively low virulence) for comparative investigation of splenic immune profiles during acute infection stages, focusing on B cells, GCs, and myeloid cells. We provided strong evidence for Karp strain virulence-associated immune patterns, including splenic bacteremia and disease scores, GC collapse, transcriptional signatures associated with inflammatory responses, and myeloid cell influx and activation. Conversely, Gilliam-infected mice developed adequate IgM and IgG responses to TSA56 (*Ot* major surface proteins) as infection progress, formed organized GC structures during infection, activated transcriptomic pathways, with regulated myeloid cell responses. Collectively, this pioneer study helps define shared immune responses to *Ot* infection in general, and more important, reveals *Ot* strain-related alteration and activation in the context of humoral immune responses.

We noted a temporal shift in the IgG responses between Karp and Gilliam infections, which may reflect differential kinetics and composition of B cell responses. Although spleen bacterial burdens peaked at Day 8 in both models, Karp infection was consistently and significantly more severe in magnitude than that of Gilliam infection, driving stronger early immune activation, as in our previous reports (23, 25). Our finding of marked expansion of plasmablasts at Day 8 in Karp-infected mice (**Figure 4**), suggests a robust extrafollicular response that may contribute to the earlier IgG peak observed at this timepoint. In contrast, Gilliam-infected mice had elevated GC B cell responses at Day 4 (**Figure 4**), indicating early initiation of GC reactions. Furthermore, these GC B cells remained organized within GC structures in the Gilliam-infected group (**Figure 3**). While Gilliam-infect mice showed limited plasmablast expansion compared to Karp groups, these early GC responses may support sustained antibody production over time, resulting in high IgG levels by Day 12. Together, our findings suggest that Karp infection drives a rapid, plasmablast-dominated response, leading to early IgG production, whereas Gilliam infection promotes earlier GC engagement and sustained humoral responses, ultimately leading to increased IgG levels at later timepoints, despite its relatively low bacterial burdens.

The collapse of GCs has been reported during infection with *Borrelia burgdorferi*, *Salmonella typhimurium*, *Ehrlichia muris*, SARS-CoV-2, and *Plasmodium* spp (35, 36, 45, 48–50). Yet, the reported mechanisms underlying GC impairment are diverse, ranging from CCR2-dependent recruitment of SCA-1^+^ monocytes that impair GC B cell metabolism, the loss of follicular dendritic cells, CXCL13 gradient disruption by excess TNFα, and the failure to generate mature Tfh cells (35, 36, 45, 50, 51). Most, if not all, reported mechanisms are associated with uncontrolled inflammatory responses. In *Salmonella* infection, IFN-γ can induce the differentiation of SCA-1^+^CCR2^+^ monocytes, which migrate to the spleen through TNFα-mediated pathways and disrupt GC (45). Similarly, blocking TNF-α during *Ehrlichia* infection can restore splenic architecture and GC B cells (51), while *Plasmodium* and severe SARS-CoV-2 infections impair Tfh differentiation due to overproduction of TNFα and IFNγ (36, 50). Our group has reported that highly virulent *Ot* strains are associated with severe disease and excessive inflammation in murine models (23), as judged by high cell/serum levels of transcripts (*Ccl2*, *Ccl3*, *Ccl4*, *Tnf*) or proteins (MCP-1, IL-6, IL-10, IFN-γ) (23, 46). We found in **Figure 6** similar increased expression of genes associated with proinflammatory signaling pathways (*Ifng*, *Ccl2, Il1*, *Ill6*, *Tnf*) during Karp infection, which may be associated with GC collapse. More importantly, we observed a striking contrast in the influx of SCA-1^+^CCR2^+^ monocytes, which were significantly increased in Karp-infected spleens at D4 and D12 relative to the Gilliam groups (~7-fold and ~20-fold higher, respectively). Given the known role of SCA-1^+^CCR2^+^ monocytes in GC disruption (45), our finding for the persistence of SCA-1^+^CCR2^+^ monocytes in Karp infection, but not in Gilliam infection, may implicate these inflammatory monocytes in Karp infection-induced GC collapse. Likewise, our observations of Karp-infected strong influx of M1 macrophages at D12 (when bacterium burdens declined, **Figure 1**), as well as progressive influx of activated CD63^+^ neutrophils at D4-D12 (**Figure 7D**) are novel findings.

At present, neutrophil’s role in *Ot* infection has not yet been studied in detail, although we reported immune detection of neutrophils in Karp-infected spleens (23, 32). RNAseq comparison data of Karp- versus Gilliam-infected spleens revealed Karp at D4 infection-related, unique transcriptomic immune signatures for neutrophil responses (**Figure 6**), including neutrophil migration, neutrophil degranulation, and specific granule pathways. IPA analysis further revealed the involvement of neutrophils, as pathways encoding neutrophil degranulation and activation of matrix metalloproteinases were significantly upregulated in Karp infection at all time points. We also found that Karp-infected spleens showed significant upregulation of pathways involved in collagen degradation and degradation of the ECM, while Gilliam-infected spleens presented increased expression of genes encoding inhibition of matrix metalloproteases. These RNA-seq findings are consistent with flow cytometric data and suggest increased transcriptional activation of neutrophil-associated pathways during Karp infection. It is possible that neutrophil degranulation, specifically tertiary granules, plays a major role in the remodeling of splenic microarchitectures, as reported for *Trypanosoma brucei* infection (52). Upon neutrophil depletion, *T. brucei*-infected mouse survival improved, splenic plasma cell numbers increased, and ECM structure was preserved (52). Given our observation of neutrophil degranulation (high CD63 expression) in Karp infection, it will be interesting to examine if degranulating neutrophils play a role in destruction of ECM noncellular scaffolding, abrogation of splenic MZs, and the collapse of GCs.

Spleen compartmental organization of the marginal zone and white pulp is critical to its function, serving as the first line of defense against blood-borne pathogens (29). The splenic MZ is next to the white and red pulp in the spleen, allowing MZ cells to capture blood-borne pathogens and initiate adaptive immune responses in the white pulp, including GC responses (29, 42, 43). We found a distinctive feature of severe infection caused by Karp strain, the abrogation of splenic MZs. Confocal imaging revealed that macrophage populations of MZ disappeared, leaving the MZ border nearly absent at D12. Flow cytometry further confirmed that the MZ B cell population was reduced at D8 and D12 of Karp infection. Given the importance of the MZ in GC responses, the abrogation of this region may be a contributing factor for GC collapse during Karp infection. While we have not yet identified the duration of GC disorganization or MZ disappearance, the loss of these structures may impair the development of adaptive immune responses to other pathogens (45).

While spleen is one of the target organs for *Ot* infection, it remains unclear as to how *Ot* establishes its infection in the spleen (46) and what cell types are its initial targets (37). Since MZ is designed for phagocytic cells to encounter blood-borne pathogens, it is possible that *Ot* may enter the spleen via the blood and establish infection in the phagocytic cells of the MZ. This has been shown for other intracellular pathogens such as *Listeria monocytogenes*, which metallophilic macrophages upon entry into the spleen (53–55). Given the phagocytic cell tropism of *Ot*, our findings of abrogated splenic MZ warrant further investigation of the role of MZ as an early *Ot* target.

In this study, we observed progressive changes in splenic white pulp during Karp or Gilliam infection (**Figure 3**). These changes included shrunken white pulp regions and T cell localization outside of T cell zones. A similar observation of shrunken white pulp was observed during *Salmonella* infection in mice, which took seven weeks for typical white pulp architecture to be restored (35). Disturbance of MZ and metallophilic macrophage populations that occurred during acute *Salmonella* infection were still unrestored after seven weeks, unlike splenic white pulp (35). Since we observed perturbations in the splenic white pulp regardless of strain or disease severity, it is likely that *Ot*, irrespective of virulence, can alter the microarchitecture of the spleen. While a previous study deemed humoral immunity noncritical for *Ot* clearance (56), it remains unclear whether this abrogation in humoral immunity relates to immune evasion of *Ot*. It is yet to be determined whether the disorganization of the splenic microarchitecture is favorable for host by increasing immune cell-*Ot* interactions, or for *Ot* to divert the development of humoral immune responses to persist in the spleen. Nevertheless, our findings suggest that splenic reorganization and inflammation during *Ot* infection may be necessary to clear infection, but it may come at the expense of humoral immune responses and increased immunopathogenesis.

This study has several limitations, some of which warrant further investigation. Firstly, our observation of GC collapse and MZ abrogation at acute Karp infection would be strengthened with long-term studies (a few weeks or months post-infection). Such studies will reveal if lymphoid structures will be recovered at the convalescence stages. Secondly, given that intradermally-inoculated Karp bacteria do not induce severe scrub typhus outcomes in either wild-type B6 or outbred CD-1 mice (30, 57), the use of knockout mouse models is an alternative approach to define Karp- versus Gilliam-induced immune responses in skin-draining lymph nodes and other lymphoid organs (25, 39). Future research involving such knockout mice would help examine the secondary lymphoid organs (draining lymph nodes, etc.) to further define whether Karp strain disrupts lymphoid architecture or impairs humoral immune responses in other tissues. Future investigation of antibody secreting cell responses from draining or distal lymph nodes will be of great value to better understand sera IgM and IgG. Thirdly, our findings of differential transcriptional signatures would be strengthened with additional research at the protein level for cytokines/chemokines and B cell function-related proteins. Further examination of the biological roles of antibodies between two infection models will be useful. Given our observations that both *Ot* strains altered splenic white pulp architectures and T cell zones, it will be important to further examine shared and *Ot* strain-specific responses at the single-cell levels. While this study is focused on primary infection, an important consideration is secondary exposure with homologous and heterologous strains, given that scrub typhus immunity is known to be short-lived (15, 16). Further research into immune landscapes upon homologous and heterologous infection will be important for determining how immunological memory shapes strain-specific responses, tissue remodeling pathways, and the durability of protective immunity.

In summary, this is the first study that uncovers an *Ot* virulence-associated trend for impaired GC and humoral immune responses during severe infection, which may be linked to enhanced inflammatory transcriptional signatures and innate immune cell infiltration. Karp-induced severe disease (high splenic bacterial burden, M1 and neutrophil influx and activation) was positively correlated to MZ perturbation and GC disruption, whereas Gilliam-induced (self-limiting) infection induced adaptive and balanced cellular and humoral immune responses. Our study has provided unique insights into the abrogation of humoral immune responses during severe *Ot* infection and showed evidence of an inflammation-related mechanism that has been demonstrated to provoke GC collapse and disruption of splenic microarchitecture in other infection models. This study helps fill the knowledge gap as to how *Ot* strain virulence influences the acute adaptive immune responses to infection.

## Data Availability

The RNA sequencing data generated in this study have been deposited in the Gene Expression Omnibus (GEO) under accession number GSE290855. Other datasets generated and analyzed during this study are available from the corresponding author upon request.
