# Supplementary material for: Differential spleen immune signatures and germinal center responses during acute infection with Orientia tsutsugamushi Karp versus Gilliam strains
Source: Front Immunol. 2026 Apr 23;17:1791779. doi: 10.3389/fimmu.2026.1791779 (PMC13149155; doi:10.3389/fimmu.2026.1791779)

**S1 Table- Primer sequences for qRT-PCR analysis used within the study.**

| Gene Target | Forward 5’-3’ | Reverse 5’-3 |
| --- | --- | --- |
| *Ccr7* | TGTACGAGTCGGTGTGCTTC | GGTAGGTATCCGTCATGGTCTTG |
| *Cd40* | TGTCATCTGTGAAAAGGTGGTC | ACTGGAGCAGCGGTGTTATG |
| *Cd86* | TGTTTCCGTGGAGACGCAAG | TTGAGCCTTTGTAAATGGGCA |
| *Cxcr5* | ATGAACTACCCACTAACCCTGG | TGTAGGGGAATCTCCGTGCT |
| *Icosl* | TAAAGTGTCCCTGTTTTGTGTCC | ATTGCACCGACTTCAGTCTCT |
| *SLAMF1* | CAGAAATCAGGGCCTCAAGAG | CACTGGCATAAACTGTGGTGG |
| GAPDH | TGGAAAGCTGTGGCGTGAT | TGCTTCACCACCTTCTTGAT |
| *O. tsutsugamushi* 47-kDa | AACTGATTTTATTCAAACTAATGCTGCT | TATGCCTGAGTAAGATACTGTAATGGA |

**S1 Figure-** **Serum IgM and IgG OD curves from Karp- vs. Gilliam-infected mice.** Mice were infected, as described in Fig 1. Infected and mock serum samples (5 per group) were serially diluted at 1:3 ratios. Antibodies specific to TSA56 (both Karp and Gilliam recombinant proteins, 1:1 mixed) were measured via indirect ELISA assays for IgM responses and IgG responses, respectively.

**
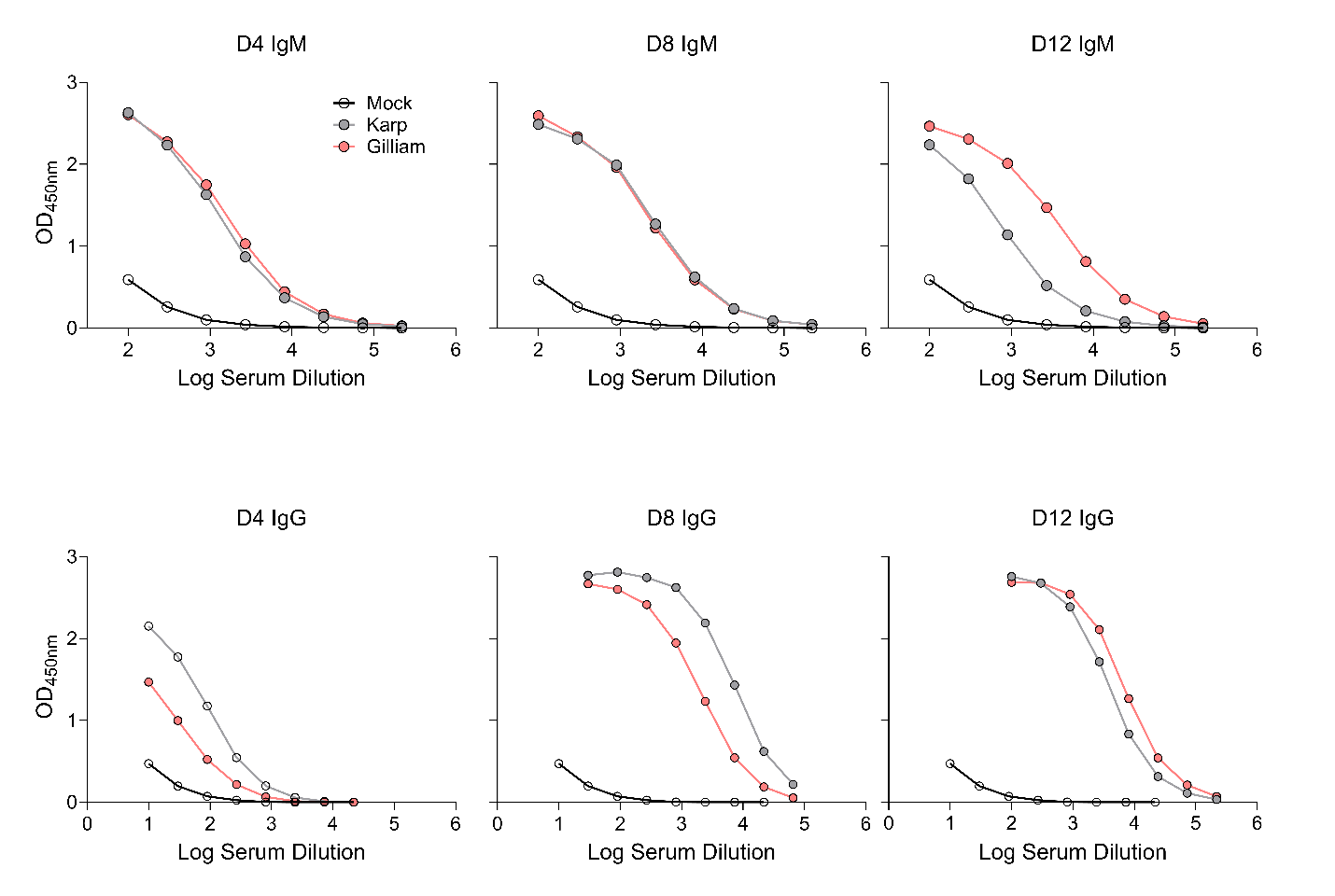
**

**S2 Figure- Equivalent absolute cell numbers of T cell subsets in infected spleens.** Mock and infected mice were euthanized, and their spleens were harvested at indicated timepoints (n = 5 per group), as described in Fig. 1. Single-cell suspensions were prepared and stained for indicated cell surface markers and gated, as described in Fig. 4. Flow cytometric analysis was performed, and results were presented as absolute numbers of various splenic T cell populations. Data are shown as mean ± SEM from a single experiment and are representative of two independent experiments with similar trends. For statistical analysis, one-way ANOVA was used with a Tukey’s multiple comparisons test for comparison of Karp- and Gilliam-infected samples at each timepoint. Asterisks were representative of comparison between Karp- and Gilliam-infected mice at each timepoint. *, p <0.05; **, p <0.01; ***, p <0.001; ****, p <0.0001.


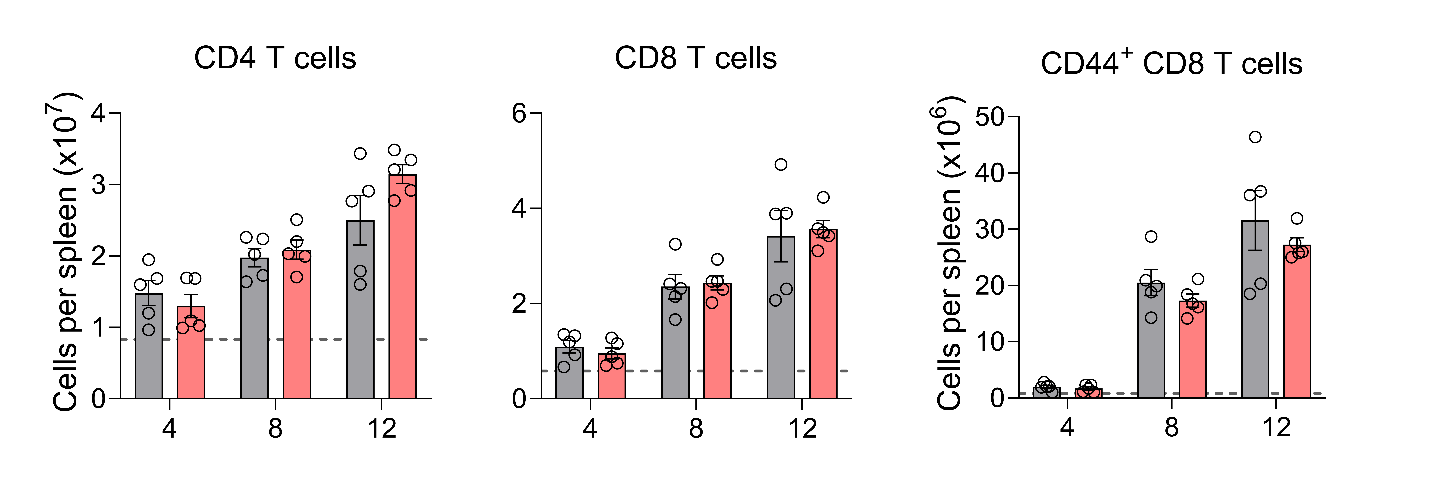


**S3 Figure- Overview of differentially expressed genes identified by RNAseq for Karp vs. Gilliam comparisons.** Mice were infected, as described in Fig. 1; RNA was isolated from spleen tissues of infected mice and sent for RNAseq analysis by Novogene (n = 4 per group). Data were normalized and differentially expressed genes were identified by using the DESeq2 R library. Principal Component Analysis was performed on the FPKM expression values (A). Differentially expressed genes detected by Karp versus Gilliam comparisons were done at D4, D8, and D12 on all samples and plotted (B).

A

**
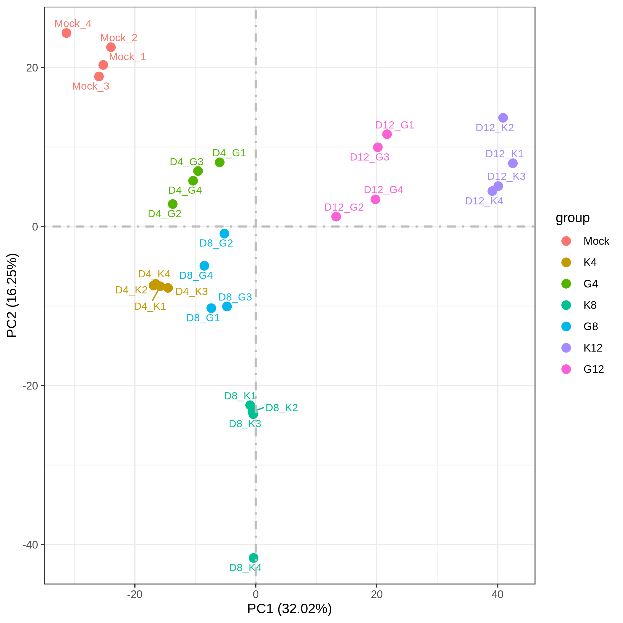
**

**
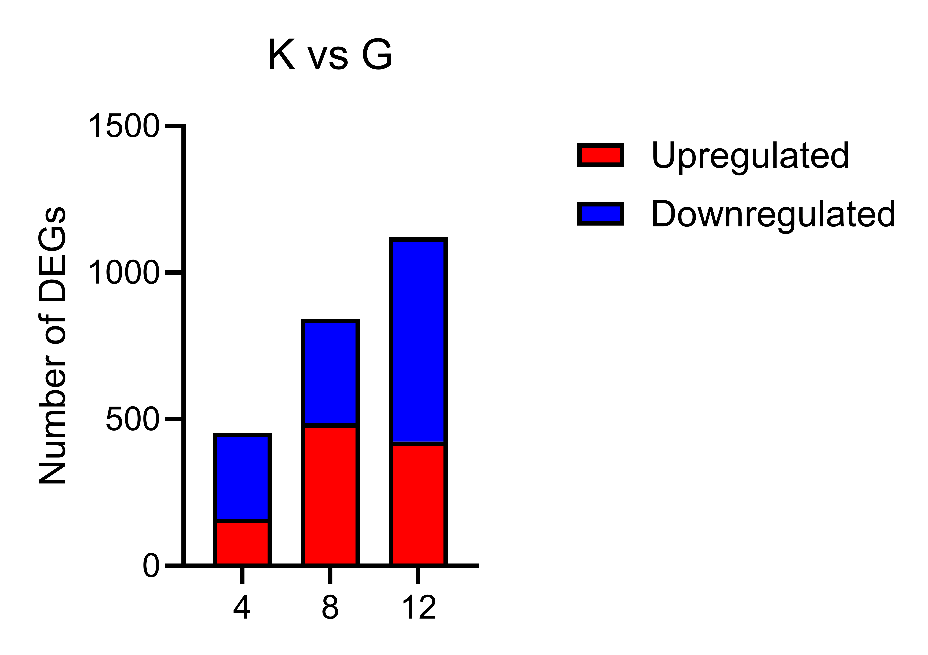
**

B

**
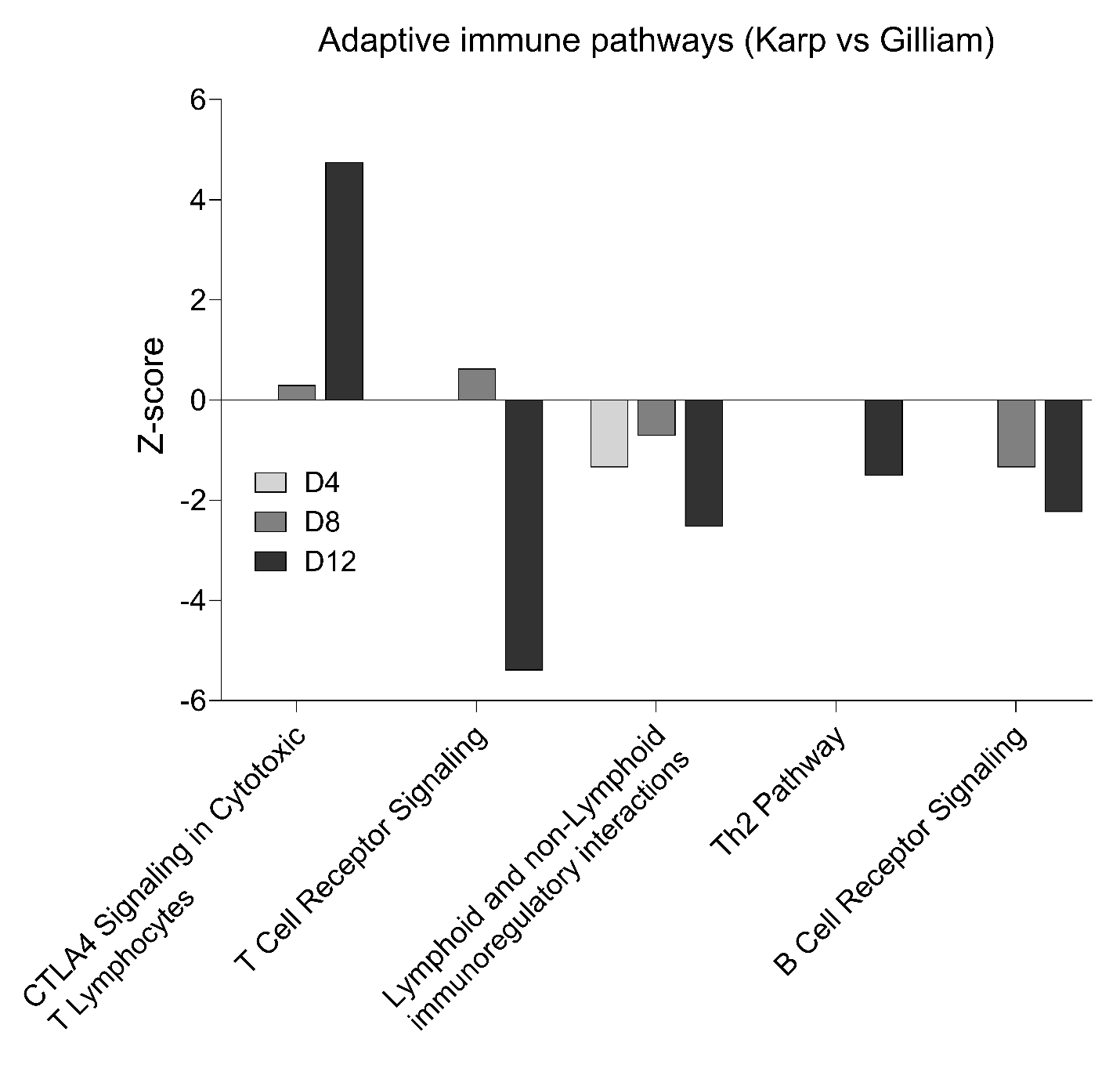
S4 Figure- B cell activation differentially expressed genes from RNAseq for Karp vs. Gilliam comparison at D12.** Mice were infected, as described in Figure 1; RNA was isolated from spleen tissues of infected mice and sent for RNAseq analysis by Novogene (n = 4 per group). Data were normalized and differentially expressed genes were identified using DESeq2 R library. B cell activation gene list was downloaded from Gene Ontology and Biological Process. Differentially expressed genes from B cell activation gene list were plotted and shown in heatmap from Karp vs. Gilliam comparison at D12 (A).

A


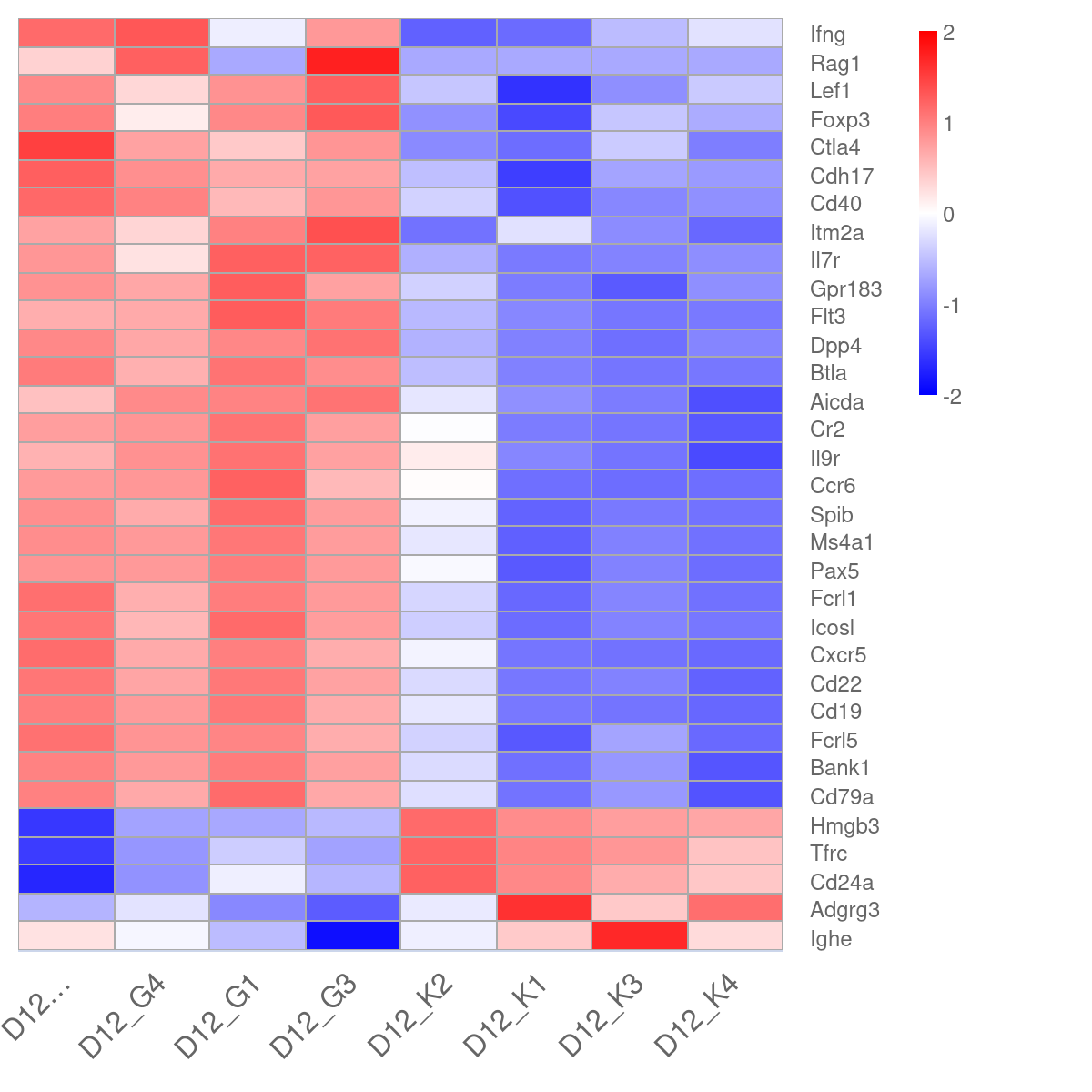


Gilliam

Karp

1 2 3 4 1 2 3 4

B

**S5 Figure- *Mmp8* and *Mmp9* differential expression from RNAseq.** Mice were infected, as described in Figure 1; RNA was isolated from spleen tissues of infected mice and sent for RNAseq analysis by Novogene (n = 4 per group). Differentially expressed genes from D4 Karp vs. Mock, D4 Gilliam vs. Mock, D8 Karp vs. Mock, D8 Gilliam vs. Mock, D12 Karp vs. Mock, and D12 Gilliam vs. Mock comparisons were identified. Select matrix metalloprotease-encoding genes were plotted using log2-fold change and displayed.


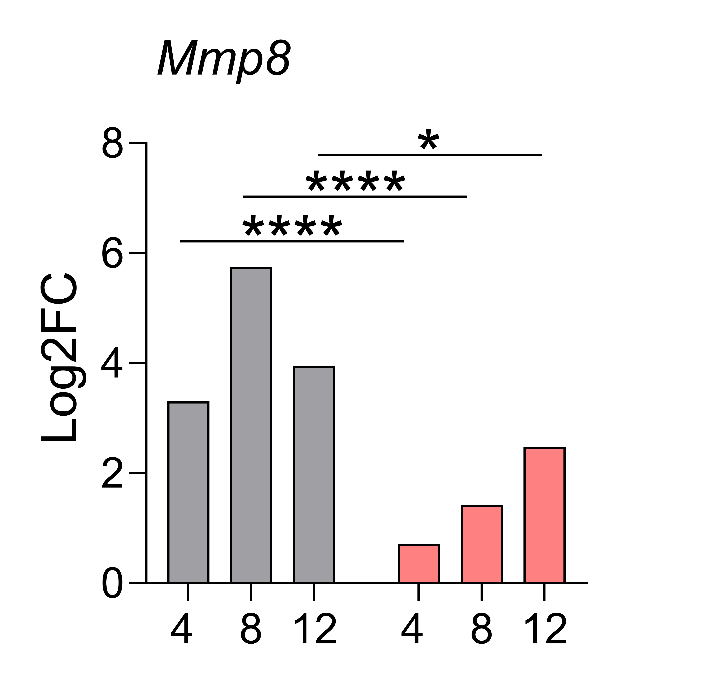

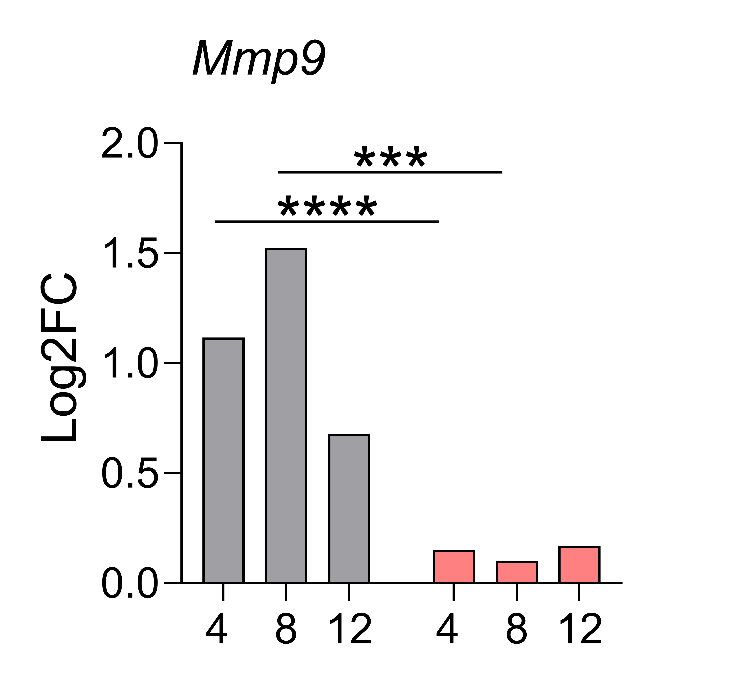


**S6 Figure- Differentially expressed inflammation-related genes between Karp versus Gilliam comparisons from RNAseq.** Mice were infected, as described in Figure 1; RNA was isolated from spleen tissues of infected mice and sent for RNAseq analysis by Novogene (n = 4 per group). Data were normalized and differentially expressed genes were identified using DESeq2 R library. Differentially expressed inflammation-related genes, shown as Log2FC, between Karp versus Gilliam comparisons at different timepoints were plotted and shown in heatmap.


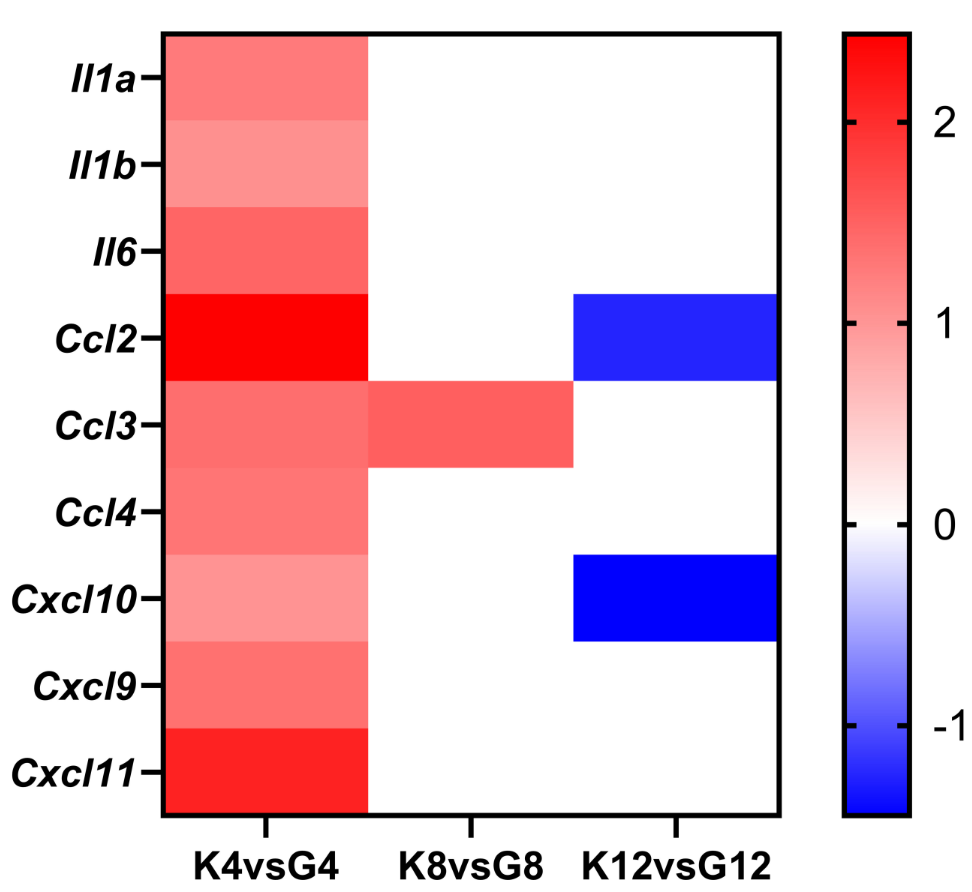

Supplement: Supplementary file 1 [file DataSheet1.docx]
